# SEG-SLAM: Dynamic Indoor RGB-D Visual SLAM Integrating Geometric and YOLOv5-Based Semantic Information

**DOI:** 10.3390/s24072102

**Published:** 2024-03-25

**Authors:** Peichao Cong, Jiaxing Li, Junjie Liu, Yixuan Xiao, Xin Zhang

**Affiliations:** School of Mechanical and Automotive Engineering, Guangxi University of Science and Technology, Liuzhou 545006, China

**Keywords:** dynamic environments, RGB-D VSLAM, YOLOv5, semantic segmentation

## Abstract

Simultaneous localisation and mapping (SLAM) is crucial in mobile robotics. Most visual SLAM systems assume that the environment is static. However, in real life, there are many dynamic objects, which affect the accuracy and robustness of these systems. To improve the performance of visual SLAM systems, this study proposes a dynamic visual SLAM (SEG-SLAM) system based on the orientated FAST and rotated BRIEF (ORB)-SLAM3 framework and you only look once (YOLO)v5 deep-learning method. First, based on the ORB-SLAM3 framework, the YOLOv5 deep-learning method is used to construct a fusion module for target detection and semantic segmentation. This module can effectively identify and extract prior information for obviously and potentially dynamic objects. Second, differentiated dynamic feature point rejection strategies are developed for different dynamic objects using the prior information, depth information, and epipolar geometry method. Thus, the localisation and mapping accuracy of the SEG-SLAM system is improved. Finally, the rejection results are fused with the depth information, and a static dense 3D mapping without dynamic objects is constructed using the Point Cloud Library. The SEG-SLAM system is evaluated using public TUM datasets and real-world scenarios. The proposed method is more accurate and robust than current dynamic visual SLAM algorithms.

## 1. Introduction

In recent years, significant progress has been made in mobile robot localisation, mapping, and autonomous navigation technologies [1]. In particular, simultaneous localisation and mapping (SLAM) [2,3], which uses data from on-board sensors to achieve localisation and mapping of environments without prior information, has become indispensable fundamental technology for mobile robots. SLAM can be divided into laser and visual SLAM according to the sensor type [4,5,6]. Laser SLAM is currently the most stable and mainstream method for localisation and navigation. However, it is limited by poor recognition of certain object categories and high costs. Compared to laser SLAM, visual SLAM is more flexible and provides rich semantic information, which makes it suitable for complex and diverse environments. Therefore, visual SLAM is gradually gaining popularity, and it has become a research hotspot.

In visual SLAM, the main sensor is a camera. Common types of cameras include monocular, stereo, and red/green/blue–depth (RGB-D) cameras. As visual SLAM has developed [7], the localisation and mapping technology for certain scenes has matured. Moreover, many excellent visual SLAM systems have been proposed, including orientated FAST and rotated BRIEF (ORB)-SLAM1-3 [8,9,10], visual-inertial system (VINS)-Mono [11], large-scale direct (LSD)-SLAM [12], directed sparse odometry (DSO) [13], and parallel tracking and mapping (PTAM) [14]. However, although existing visual SLAM systems have achieved excellent performance for certain scenes, some problems remain to be solved. For example, most visual SLAM algorithms rely heavily on the environment being static, where changes in the field of view mainly arise from the motion of the camera, and there is a lack of other dynamic objects in the environment. However, dynamic objects are indispensable in real environments, and their presence can cause erroneous data associations in the feature point matching process, which affects the localization and map-construction accuracy.

The existence of dynamic objects is not unique in form. Dynamic objects studied in this paper are divided into two categories based on their motion state: obviously dynamic objects, such as walking people, and potentially dynamic objects, such as movable chairs. When dynamic objects are present, traditional visual SLAM algorithms cannot satisfy localisation and mapping requirements. This paper categorizes dynamic visual SLAM solutions into two types: geometric [15,16,17,18,19] and semantic [20,21,22,23,24,25] methods. Geometric methods use the geometric information measured by sensors to detect and reject dynamic objects, whereas semantic methods use neural networks. Geometric methods have higher computational efficiency in known environments and they can satisfy real-time requirements. However, they cannot detect potentially dynamic objects because they do not have access to prior information about the scene. By contrast, semantic methods provide a deeper understanding of the environment and more information about objects. However, they rely heavily on the quality of the underlying neural networks and cannot guarantee real-time while satisfying accuracy requirements. To improve the robustness of visual SLAM systems in dynamic scenes and improve the comprehension of semantic information, researchers have begun to combine geometric and semantic methods. However, two significant issues remain unresolved.

First, when obtaining the a priori information of dynamic objects, the target detection method is unable to accurately obtain the contour information of dynamic objects due to the inclusion of background points in the detection frame, and the semantic segmentation method can accurately segment the contour, but it relies heavily on the quality of the neural network, which affects the real-time performance of the system. Second, when removing dynamic feature points, if the quality of the neural network is poor, the error of the mask edge points is significant, and the feature points of potentially dynamic objects cannot be judged solely on the basis of the information from the neural network, as they are at rest in some frames.

Therefore, to address these issues, this study presents an indoor dynamic visual SLAM system called SEG-SLAM, which is based on the ORB-SLAM3 framework. You only look once (YOLO)v5 target detection and semantic segmentation algorithms, as well as the epipolar geometry method are introduced to achieve recognition and rejection of dynamic feature points. The system reduces interference from dynamic objects and uses semantic information to reconstruct the static background, which improves the localisation and mapping accuracy. The main contributions of this paper are summarized as follows:

(1) Aiming at the dual challenges of accuracy and real-time performance in obtaining a priori information about dynamic objects, this paper builds a fusion module of target detection and semantic segmentation using the YOLOv5 deep-learning method based on the ORB-SLAM3 framework, which generates basic semantic information of dynamic objects in a scene in real time.
(2) Aiming at the complexity of dynamic feature point rejection, this paper designs differentiated dynamic feature point rejection strategies. Among them, obviously dynamic objects combine depth information to judge dynamic feature points, and potentially dynamic objects utilize the epipolar geometry method to judge dynamic feature points. This strategy reduces the effects of dynamic objects and improves the localisation and mapping accuracy.
(3) Aiming at the problem of dynamic feature points affecting dense map construction, this paper constructs dense 3D maps with static points after removing dynamic feature points. The SEG-SLAM system is evaluated in both the Technical University of Munich (TUM) RGB-D dataset and real dynamic scenes. Experimental results show that the SEG-SLAM system outperforms most mainstream dynamic visual SLAM algorithms.

The rest of this paper is organized as follows: Section 2 discusses related work. Section 3 introduces the SEG-SLAM system framework in detail and describes the proposed method. Section 4 evaluates the algorithm of this paper in the TUM datasets and real scenes. Section 5 and Section 6 are the discussion and conclusion of this paper.

## 2. Related Work

In recent years, many researchers have attempted to improve the accuracy and robustness of visual SLAM systems for dynamic scenes. Consequently, the detection and rejection of dynamic features has emerged as a promising solution. Detection and rejection methods can be divided into three categories: geometric, semantic, and geometric and semantic fusion methods.

### 2.1. Geometric Methods

Geometric methods estimate the poses of objects without considering prior information about the scene. This approach assumes that only static features can satisfy the geometric constraint model, which utilizes the characteristics of dynamic objects against a static background. In 2015, Bakkay et al. [15] developed an enhanced scene-flow approach for detecting dynamic objects. This approach uses a regional growth segmentation algorithm to separate the dynamic and static parts of a scene, which prevents mismatches during the alignment step. However, the pixel motion cannot be estimated accurately when an object moves rapidly or exhibits motion blur. In 2017, Li et al. [16] used deep-edge points to establish corresponding relationships and devised a static weighting approach to add static weights to the intensity-aided iterative closest points (IAICP) approach for alignment tasks, which reduced the impact of dynamic feature points. This method can effectively extract foreground edge points, although the extraction accuracy for edge points under large occlusions is relatively low. Therefore, significant errors will occur during the extraction of deep edge points in scenarios without rich geometric information.

In 2019, Wang et al. [17] used fundamental matrix detection to cluster depth images based on inconsistent feature points. When the outliers in clustered regions exceed a predefined threshold, the region is identified as a dynamic region. This information is integrated into the visual SLAM system as part of its data filtering. However, this method has high computational costs and cannot handle potentially dynamic objects. In 2021, Long et al. [18] proposed a method that assumed that all dynamic objects were single rigid bodies and concurrently segmented and tracked both static and dynamic objects. This system can simultaneously localize and reconstruct both static backgrounds and rigid dynamic components in environments with large occlusions caused by dynamic objects. However, it cannot segment, track, and reconstruct multiple rigid targets. In 2022, Ni et al. [19] suggested the notion of feature point reliability markers, which dynamically control the number of feature points within a predefined range. They used a frame transfer method for dynamic object detection and introduced a new fault-handling mechanism to improve the localisation and mapping accuracy. However, all geometric methods ignore potentially dynamic objects, which can cause erroneous data associations.

### 2.2. Semantic Methods

Owing to developments in neural-network technology, the number of semantic methods being applied to visual SLAM systems has increased considerably in recent years. Advanced deep-learning techniques [20] are typically used to generate semantic information for object detection and semantic segmentation. Tunio et al. [21] used the U-Net architecture based on semantic segmentation for target detection and location, achieving high accuracy. Wang et al. [22] used the fully convolutional instance-aware semantic segmentation (FCIS) [23] algorithm to calculate the image boundary boxes for an input RGB image and obtained the mask information for the entire image. Predefined candidate categories were selected from the Microsoft common objects in context (MS COCO) dataset and the results were input into the visual SLAM system in the form of masks.

Yuan et al. [24] proposed the semantic and depth (SaD)-SLAM system, which combines semantic and depth information to extract feature points of dynamic objects and detect their states. The camera pose is calculated using static feature points extracted from dynamic objects and feature points extracted from static objects. For the semantic information component, they employed the Mask R-CNN method to extract image information. Bescos et al. [25] used the Mask R-CNN for instance segmentation with visual feature tracking of dynamic objects. They optimized the structure of static scenes and dynamic objects based on the trajectories of the camera and motion agent. Fang et al. [26] proposed using the Mask R-CNN to extract semantic information to construct semantic descriptors and utilized a knowledge graph to determine prior motion relationships between entities. This facilitates the rejection of dynamic objects and improves the tracking and localisation accuracy of robots in dynamic scenes. However, semantic methods rely heavily on the quality of the neural networks, which makes it difficult to satisfy the requirements for both real-time application and accuracy.

### 2.3. Geometric and Semantic Fusion Methods

Geometric methods have significant advantages in dealing with conventional dynamic objects. However, they cannot be used to accurately identify and reject dynamic features owing to insufficient prior information for potentially dynamic objects. Semantic methods are well suited to scenes with predefined dynamic objects. However, there are poorly suited to general dynamic scenes as they cannot detect objects beyond the scope of the training set. Geometric and semantic approaches may be combined to overcome their respective limitations, and fusion methods may offer more comprehensive and reliable solutions for dynamic object detection.

In 2018, Bescos et al. [27] proposed the DynaSLAM system, leveraging the Mask R-CNN segmentation and multiview geometry methods to detect and reject dynamic features in the RGB-D mode. However, the system involves a complex network model and low real-time performance. In 2022, Zhong et al. [28] proposed the weighted features (WF)-SLAM system that closely integrated semantic information and geometric dynamic object detection algorithms to obtain accurate dynamic object information for a scene. Dynamic information is used to initialise the system and define the feature point weights, which transforms the pose optimisation of the ORB-SLAM2 system into a weighted joint optimisation system. However, the relatively protracted execution time of this method may pose a concern. Wu et al. [29] introduced the YOLO-SLAM system, utilising a lightweight target detection network called Darknet19-YOLOv3. This network uses low-latency backbone acceleration and generates basic semantic information for visual SLAM systems. Wu et al. also introduced a novel geometric constraint approach to exclude the dynamic features within the detection zone and used the depth difference method based on random sample consensus (RANSAC) to distinguish dynamic features. However, the performance of the system can be affected by the rotation of the camera. Xu et al. [30] introduced the efficient semantic dynamic (ESD)-SLAM system, employing a lightweight semantic segmentation network, the fully convoluted harmonic densely connected network (FcHarDNet), to extract semantic information and the region-growing algorithm to optimise the semantic segmentation boundaries. By combining semantic information with multiview geometric information, dynamic features are rejected effectively, which further improves the localisation accuracy. However, this method can result in excessive image segmentation. You et al. [31] proposed the multimodal semantic (MISD)-SLAM system designed for dynamic environments, which involves instance segmentation, dynamic pixel rejection, and semantic 3D mapping construction. The instance segmentation network provides semantic information about the surrounding environment at the instance level, which is combined with the multiview geometric constraints and K-means clustering algorithm to further improve the positioning and mapping accuracy for dynamic scenes. However, this method does not consider the detailed treatment of dynamic objects that may appear to be static in some frames.

In 2023, Zhang et al. [32] introduced a visual SLAM system that utilizes the YOLOv5s CNN and Ghostnet backbone network to detect dynamic objects in a scene and integrates the coordinate attention mechanism to improve the small-target recognition accuracy. However, the method used to reject dynamic objects is relatively simple and it rejects all the points within the detection box without fully considering the background points within the detection box. Cheng et al. [33] introduced the SG-SLAM system, using a normalized CNN (NCNN) as the basic framework for object detection threads, single-shot detector as the detection head, and MobileNetV3 as the feature extractor to detect dynamic objects in a scene. The system uses epipolar geometric constraints to screen dynamic and static objects in the geometric part. However, this method fails when the dynamic objects move along the epipolar lines. Song et al. [34] proposed the SCE-SLAM system, which introduces an object detection thread, self-motion estimation module, and dynamic feature point rejection module to the ORB-SLAM3 framework. During the object detection thread, the YOLOv7 neural network is used to detect dynamic objects. However, this method does not consider potentially dynamic objects. Similarly, Jin et al. [35] proposed a visual SLAM system that added semantic segmentation, dynamic feature rejection, and dense point cloud mapping to the ORB-SLAM3 framework. The SparseInst segmentation network is used in the semantic segmentation thread to detect dynamic objects, and the epipolar geometry approach is used in the dynamic feature rejection module to reject potentially dynamic feature points. However, the real-time performance of the system is influenced by the computational demands associated with the semantic segmentation network.

At present, researchers generally prefer to use geometric and semantic fusion methods to solve the problem of dynamic objects in visual SLAM systems. Target detection and semantic segmentation methods are dominant in deep learning, whereas multiview geometry and optical flow methods are dominant in geometry. However, concerning real-time performance and stability, integrating these approaches for practical applications still poses significant challenges. For example, semantic segmentation algorithms have complex networks that have long processing times, whereas target detection algorithms often fail to represent object boundary information clearly. Therefore, this study builds a fusion module of target detection and semantic segmentation using the YOLOv5 deep-learning method based on the ORB-SLAM3 framework with high precision and robustness.

## 3. Methods

In this section, the SEG-SLAM system in the RGB-D mode is introduced in terms of dynamic feature extraction, dynamic feature point rejection, and dense 3D mapping. First, the overall framework of the SEG-SLAM system is described in detail. It consists of five main parallel threads, the (1) tracking thread, (2) fusion thread for target detection and semantic segmentation, (3) local mapping thread, (4) loop closing thread, and (5) dense 3D mapping thread. Threads 1, 3, and 4 are the three main threads of the ORB-SLAM3 framework and threads 2 and 5 are two new threads introduced in this study. The YOLOv5 depth learning method is implemented in thread 2 and dense 3D mapping is implemented in thread 5. Other methods are implemented in the tracking thread. Second, the dynamic features in a scene are identified using the fusion method of target detection and semantic segmentation. Third, a dynamic feature point rejection strategy is proposed based on prior, depth, and geometric information. Finally, a static dense 3D mapping is constructed based on semantic and geometric information.

### 3.1. SEG-SLAM Framework

**Figure 1:**
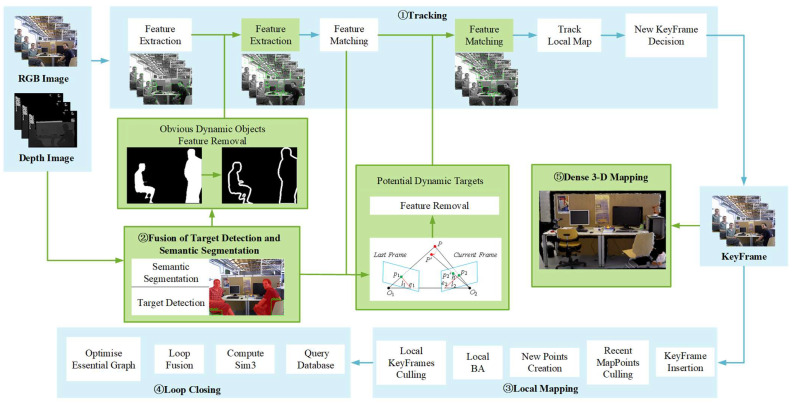
SEG-SLAM framework. This study introduces new fusion and dense 3D mapping threads (indicated in green) to the ORB-SLAM3 system.

ORB-SLAM3 is an advanced visual SLAM system that is robust for static scenes. However, it cannot provide accurate estimates for the camera pose or provide stable maps for dynamic scenes owing to the effects of dynamic objects. Therefore, this study aims to optimize ORB-SLAM3 to improve its robustness for dynamic scenes. Figure 1 shows the original ORB-SLAM3 system with the added fusion thread for target detection and semantic segmentation (thread 2) and dense 3D mapping (thread 5). The fusion thread for target detection and semantic segmentation relies on the YOLOv5 segmentation to provide semantic information about the dynamic objects in the scene. The tracking thread (thread 1) effectively screens and rejects dynamic feature points using the depth information, epipolar geometry, and other approaches. The dense mapping thread uses the keyframe information through the Point Cloud Library (PCL) to accurately construct a 3D dense mapping. The remaining local mapping and loop closing threads (threads 3 and 4, respectively) complete basic functions, such as updating the local map and optimising the pose graph.

During the operation of the SEG-SLAM system, each frame of the image passes through the tracking and fusion threads for target detection and semantic segmentation. In the fusion thread for target detection and semantic segmentation, the system extracts the semantic information for obviously and potentially dynamic objects from each frame. At the same time, the tracking thread also performs the task of extracting ORB feature points and screens and rejects feature points based on previously obtained semantic information. Among them, the mask boundary points are filtered based on depth information, while potentially dynamic feature points are screened utilising the epipolar geometry method. This effectively reduces the adverse effects of erroneous data associations for dynamic feature points.

### 3.2. Dynamic Object Detection

Deep learning-based target detection and semantic segmentation methods are widely used to obtain prior information about dynamic objects in a scene. Target detection methods process less information and have higher computational efficiency than semantic segmentation methods; however, they only provide bounding box information for objects and they ignore specific contours and shape information. By contrast, semantic segmentation methods can achieve fine-grained localisation at the pixel level and deal with occlusion and overlapping situations more effectively. However, they have greater computational complexity and require more computational resources. Therefore, a fusion method of target detection and semantic segmentation is utilised for the detection of each image frame. The semantic segmentation method is used to detect obviously dynamic objects, whereas the target detection method is used to detect potentially dynamic objects. This fusion method helps acquire more comprehensive prior information about dynamic objects, thereby improving the understanding and perception ability for complex scenes.

**Figure 2:**
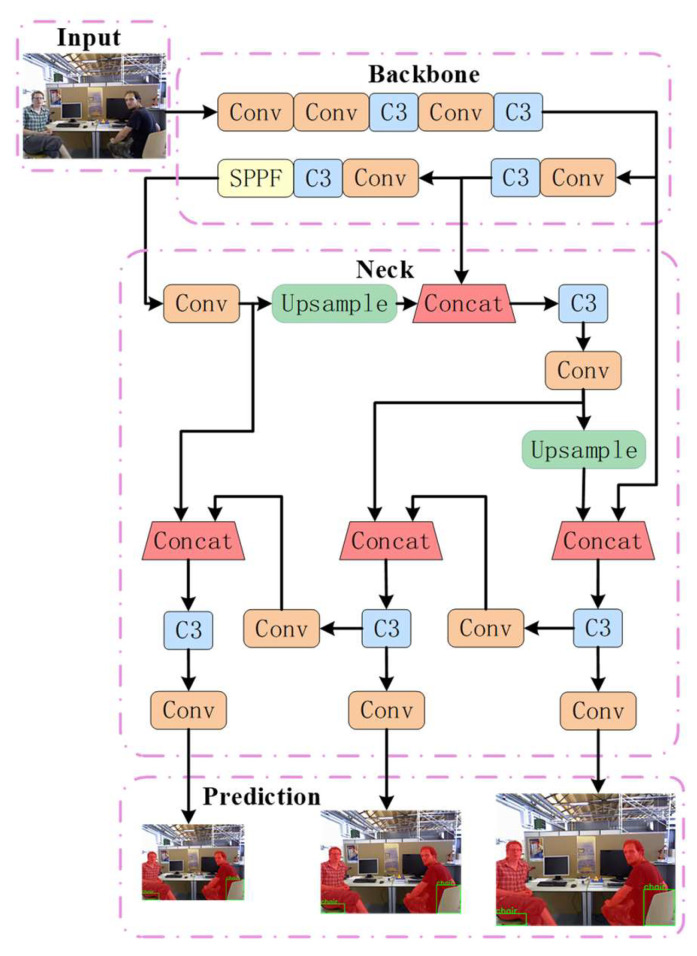
YOLOv5 network structure. Diagram showing the network structure of YOLOv5. The detection results are shown in the prediction module.

Traditional semantic visual SLAM systems face significant challenges in balancing the accuracy and real-time performance of semantic segmentation networks. Although Mask-RCNN, a common two-stage instance segmentation method, has high segmentation accuracy, it requires relatively long processing times, which makes it difficult to satisfy real-time requirements. Furthermore, some convolutional neural networks, such as fully convolutional networks (FCNs) [36] and SegNet [37] algorithms using encoder–decoder structures, have been extensively employed in visual SLAM systems. However, these approaches do not provide sufficient contextual information and only use single-scale feature maps for segmentation. Consequently, they multiscale feature fusion, which makes it difficult to accurately segment dynamic objects of different scales. To improve the accuracy and operational efficiency of the visual SLAM system, this study adopts the lightweight detection algorithm YOLOv5, and its network structure is shown in Figure 2. Among the YOLO series of algorithms, although YOLOv8 brings many innovations as an updated detection algorithm, its maturity still needs to be improved compared to other versions. Therefore, in order to ensure the stability and reliability of the detection results, this paper decides to adopt the more mature and widely verified YOLOv5 algorithm to provide the a priori information. The structure diagram is mainly divided into four modules, which are the input side of the network; the backbone network, which performs feature extraction on the input image; the neck network, which performs feature fusion on the features extracted by the backbone network; and the head output side. which has three detection heads to predict the feature map. When each frame is passed from the input to the network, the detected image can be output at the output according to the dynamic categories set in this paper. Compared to the Mask-RCNN algorithm, the YOLOv5 segmentation algorithm has a higher operational efficiency, and its design emphasises real-time performance. By integrating the target detection and segmentation tasks into a single stage, the processing speed is significantly improved. Therefore, the SEG-SLAM algorithm can satisfy the requirements for both accuracy and real-time performance.

**Figure 3:**
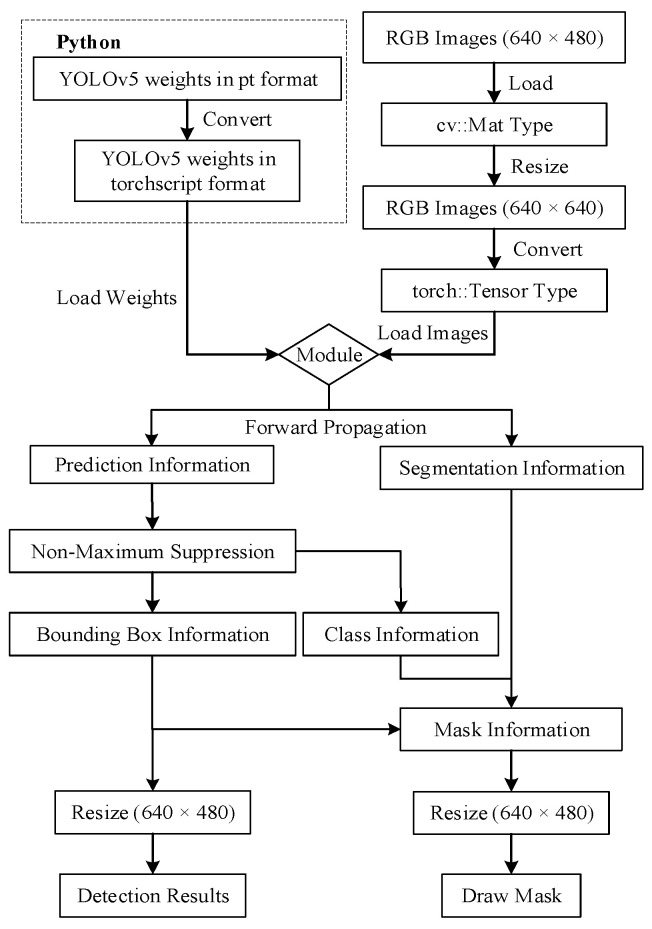
Deployment process. The section in the dashed box was coded in Python and the remainder was coded in C++.

YOLOv5 is a target-detection algorithm written in Python. To integrate it into the SEG-SLAM system more effectively, C++ is used to deploy the neural network for the YOLOv5 algorithm, as shown in Figure 3. Firstly, the trained pt weights file from the Python side is converted into a weights file in torchscript format that can be loaded by the libtorch library (the environment of Python and C++ should be guaranteed to be the same CUDA version, and also Pytorch and libtorch should be guaranteed to be the same corresponding version). Secondly, with the help of the torch::jit::load function of the libtorch library, the torchscript file is loaded to obtain the network model available for inference, and at the same time, the RGB image of the input system is converted into the torch::Tensor type and passed into the network model for forward propagation to obtain the prediction information and segmentation information, Thirdly, a non-extremely large value suppression function is written to filter the optimal detection box and the corresponding category information. Fourthly, the segmentation information is multiplied with the category information, the sigmoid activation function is applied to obtain the preliminary masking results, the masking results are adjusted according to the size of the original image, and the results are binarised. Finally, the corresponding detection box information and mask information are output, in which the results of the detection box information are the coordinates of the centre point of the detection box as well as the length and width information, and the result of the mask information is the binary image in the form of cv::Mat.

### 3.3. Dynamic Feature Point Rejection

Feature points are a set of pixels used to describe key local features in an image and they are crucial for camera pose estimation and 3D scene reconstruction. However, the presence of dynamic feature points can cause erroneous data associations in subsequent tasks, thereby affecting the accuracy of localisation and mapping. Therefore, this study adopts a feature point rejection strategy that fully integrates prior information, depth information, and epipolar geometry methods to form a tightly coupled processing pipeline. For obviously dynamic objects, the internal points are eliminated using mask information and the boundary points are assessed using depth information. For potentially dynamic objects, the dynamic attributes of the feature points (dynamic or static) are assessed using epipolar geometry methods. This strategy significantly reduces the effects of dynamic feature points on the subsequent localisation and mapping tasks, thereby improving the overall system’s accuracy and robustness.

#### 3.3.1. Mask Scaling

For obviously dynamic objects, mask information can be used to effectively improve the accuracy of dynamic feature point rejection. However, for complex dynamic objects with irregular motion or high motion uncertainty, the extraction of mask boundary information may be inaccurate and static background feature points may be included in the mask. Therefore, the mask extracted by YOLOv5 is scaled to ensure that it is fully within the range of the object (e.g., a human body). This approach aims to improve the accuracy of the mask information for complex dynamic objects by adjusting the size and shape of the mask, thereby more accurately rejecting dynamic feature points and providing more reliable data for subsequent tasks.

**Figure 4:**
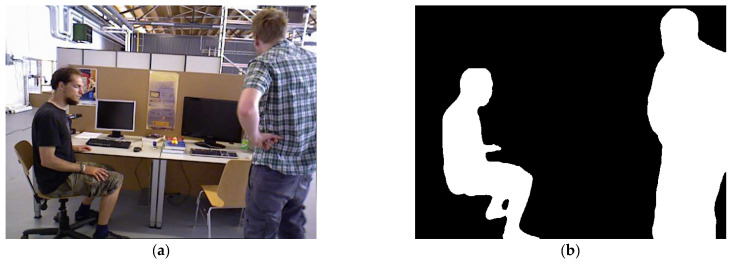
Mask information image. (**a**) Original image and (**b**) binary mask image.

**Figure 5:**
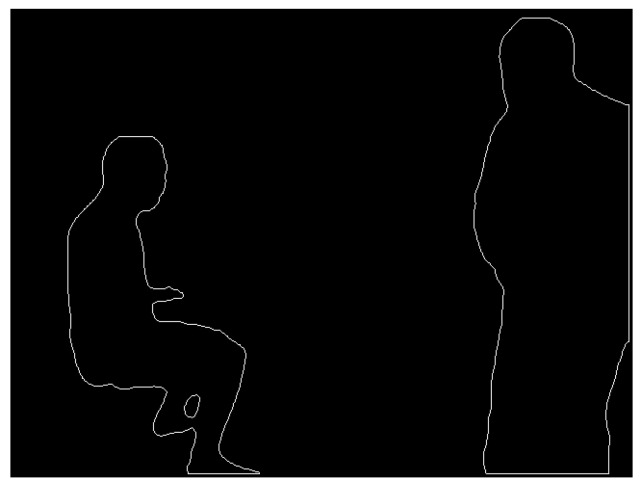
Mask contour before scaling.

The mask information for obviously dynamic objects is obtained according to the process described in Section 3.2. It consists of a binary image with pixel values of 0 or 255, as shown in Figure 4. To calculate the scaled mask region, the findContours function in the OpenCV library is used to extract the original mask contours. The contours are shown in Figure 5. The contours consist of a finite number of connected points. Therefore, we scale the pixel coordinates of these points and then use the drawContours function to draw a new binary image based on the new coordinates. The traditional coordinate scaling method calculates the relationship between the coordinate points and the centroid of the region and then scales the coordinate points along the centroid direction. However, this method has high requirements for the shape of the contour and complex shapes are difficult to process accurately. Therefore, this study presents a new approach that can be used to calculate various complex contours.

The proposed method calculates the inner and outer points at a certain distance in the normal direction from adjacent points on the original contour. One point belongs to the inward reducing contour point set and the other belongs to the outward expanding contour point set. The inward reducing and outward expanding contour point sets are denoted reducePoints and expandPoints, respectively.

Assume that two local adjacent points on the original contour have coordinates $p_{1}\left(x_{1},y_{1}\right)$ and $p_{2}\left(x_{2},y_{2}\right)$. Then, the direction vector between these two points can be expressed as

(1) $$v=p_{2}-p_{1}=\left(x_{2}-x_{1},y_{2}-y_{1}\right).$$

The normal vector coordinates are

(2) $$n=\left(y_{1}-y_{2},x_{1}-x_{2}\right)$$

and the normal coordinates of the normal vector are

(3) $$n_{e}=\left(\frac{y_{1}-y_{2}}{\sqrt{{\left(x_{1}-x_{2}\right)}^{2}+{\left(y_{1}-y_{2}\right)}^{2}}},\frac{x_{2}-x_{1}}{\sqrt{{\left(x_{1}-x_{2}\right)}^{2}+{\left(y_{1}-y_{2}\right)}^{2}}}\right).$$

Tests show that a translation distance of seven pixels produces the best scaling contour effect. The coordinates of the scaling point set are calculated using the equations

(4) $$x=x_{1}\pm \frac{7\left(y_{1}-y_{2}\right)}{\sqrt{{\left(x_{1}-x_{2}\right)}^{2}+{\left(y_{1}-y_{2}\right)}^{2}}}$$

and

(5) $$y=y_{1}\pm \frac{7\left(x_{2}-x_{1}\right)}{\sqrt{{\left(x_{1}-x_{2}\right)}^{2}+{\left(y_{1}-y_{2}\right)}^{2}}}.$$

**Figure 6:**
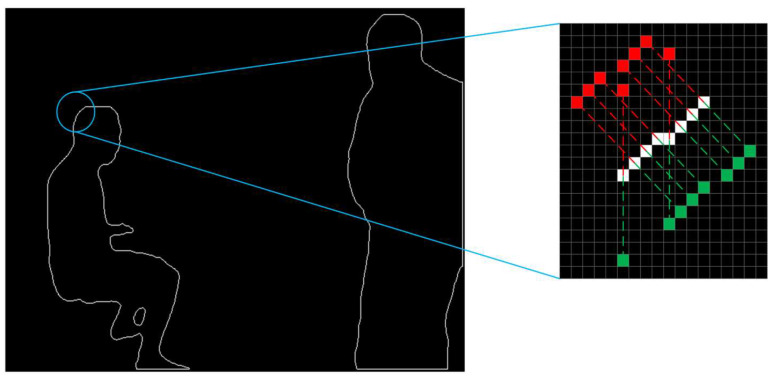
Schematic diagram showing the calculation of the translation point sets. In the enlarged image, the white, red, and green pixels represent the original, enlarged, and reduced contour point sets.

**Figure 7:**
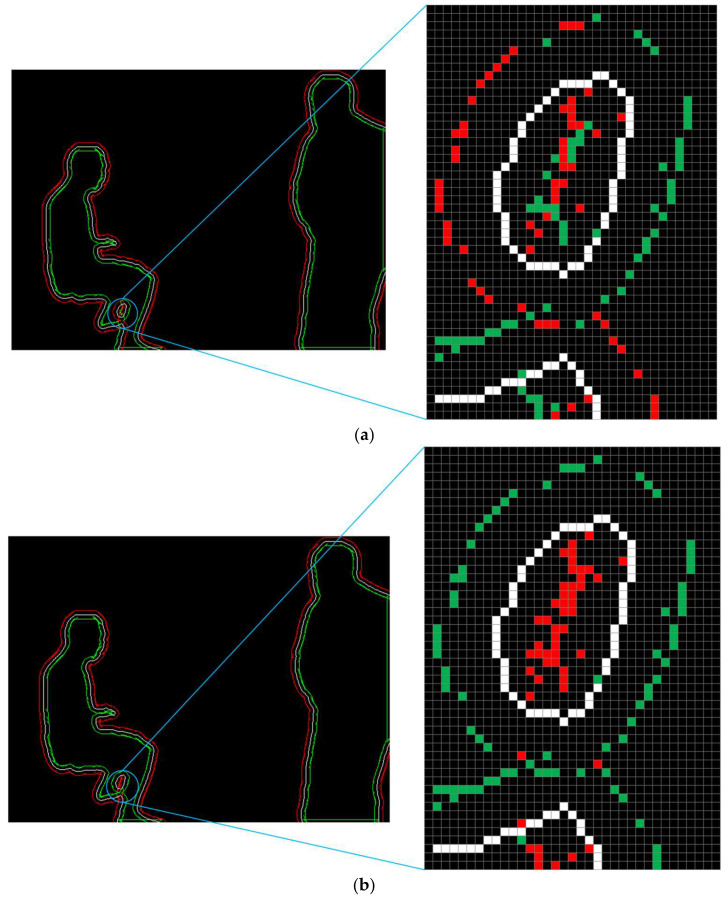
Mask contour optimisation process. The white, red, and green pixels represent the original, enlarged, and reduced contour point sets. (**a**) Process I; (**b**) Process II.

The translation point sets for the original contour in the inward and outward directions are calculated and the calculation schematic is shown in Figure 6. Subsequently, a screening strategy is used to determine which points should be included in the reduced and expanded contours. First, considering the position of the origin of the image pixel coordinate system and the principle of the findContours function, the point farthest from the origin in the first group of calculated points for each contour is added to the reducePoints point set and the other point is added to the expandPoints point set. Second, for each original contour from the 2nd to the nth point, the distances of the two calculated $\left(x,y\right)$ coordinates from the origin are compared to those of the latest coordinates in the reducePoints point set. The point with the smallest distance is added to the reducePoints point set and the other point is added to the expandPoints point set. The results are shown in Figure 7a. This strategy produces an approximate point set. However, the coordinates generated by the findContours function are all integers, which indicates that the point set is not accurate enough, particularly for the left leg of the figure.

**Figure 8:**
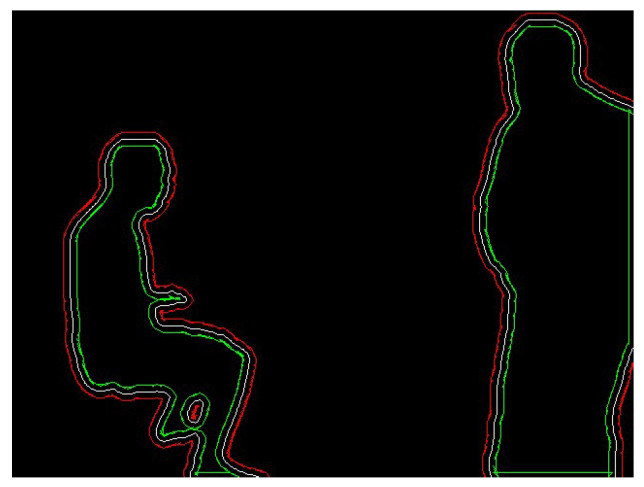
Mask contour scaling effect.

Several methods are used to eliminate incorrect points from the point set. These methods are implemented as follows:

1. If the reducePoints point set contains more than two points, the distances between the two newly calculated points *a* and *b* and the two latest points *p* and *q* in the reducePoints point set are compared. If point *a* is closer to both points *p* and *q* than to point *b*, then points *a* and *b* are added to the reducePoints and expandPoints point sets, respectively. If point *a* is closer to either point *p* or *q* than to point *b*, then the distances are compared to the previous point in the original contour. The point with the smallest distance is added to the reducePoints point set and the other is added to the expandPoints point set. The results are shown in Figure 7b. The expansion and reduction processes of the contour approximately follow the change trends of the original contour.
2. In the enlarged image on the right side of Figure 7b, it can be found that there are still individual wrong red and green dots, and some of them are too close to the white dots, and the distance is too close to achieve a better zoom effect. To further improve the calculation accuracy for the two contours, the coordinates of the 50 points before and after each point in the original contour are traversed (if there are fewer than 50 points before or after a point, the data are truncated at the beginning or end). The distances between the two newly calculated points and these points are compared. If the distance to one point is less than three, the newly calculated point is deleted from the corresponding point set. The results are shown in Figure 8.
3. A point set named newPoints is created. If both scaling points calculated for a certain point in the original contour are not deleted, then the original contour point is added to the newPoints point set. This point set is used in Section 3.3.2.

#### 3.3.2. Internal Mask Point Rejection

The mask contour is divided into inner and outer contours. As described in Section 3.3.1, the reducePoints point set is the set of points furthest from the origin. Therefore, for the outer contour, the reducePoints and expandPoints point sets correspond to inward reduction and outward expansion, respectively. By contrast, for the inner contour, the expandPoints and reducePoints point sets correspond to inward reduction and outward expansion, respectively.

**Figure 9:**
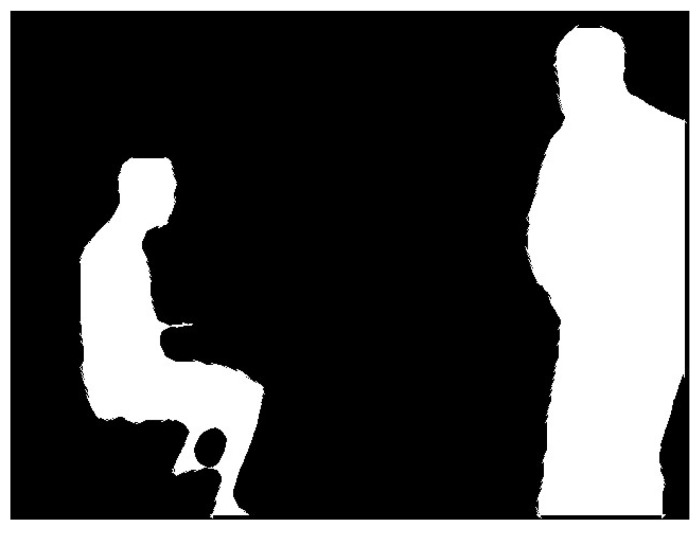
Binary image of the reduced mask contour.

**Figure 10:**
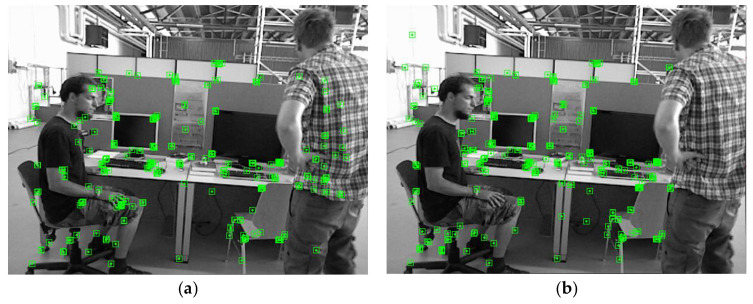
Effect of internal mask point rejection. The green points in the diagram are feature points. Points (**a**) before and (**b**) after rejection.

For the outer contour, the drawContours function is used to draw a white mask contour on a black background based on the reducePoints point set. If an inner contour also exists, the drawContours function is used to draw a black mask contour based on the expandPoints point set. This generates a new binary image, as shown in Figure 9. If the extracted feature points belong to the mask contour, they are rejected. The rejection effect is shown in Figure 10.

#### 3.3.3. Edge Mask Point Rejection

To improve the dynamic feature point rejection effect, the mask contour is reduced inwards by a certain distance, as discussed in Section 3.3.2, to ensure that all the rejected points correspond to obviously dynamic objects. However, for irregularly shaped objects, such as humans, there may be some errors in the contours identified via semantic segmentation. Therefore, the feature points located at the contour boundaries must be assessed. In this section, we will combine depth information to judge the feature points located in the intermediate region between the inwardly narrowed and outwardly expanded point sets.

**Figure 11:**
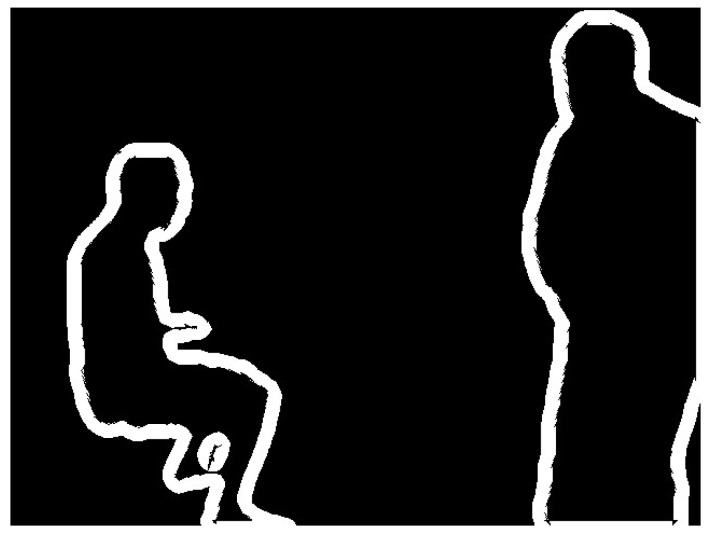
Binary mask of intermediate region.

A binary mask for the intermediate region is drawn based on the point set information obtained in Section 3.3.1. For the outer contour, the drawContours function is used to draw the expandPoints point set as white mask contours on a black background and the reducePoints point set as black mask contours. If there are inner contours, the drawContours function is also used to draw the reducePoints point set as white mask contours and the expandPoints point set as black mask contours. This generates a new binary image for the points in the intermediate mask region, as shown in Figure 11. Owing to the randomness in the movement of obviously dynamic objects, such as people, optimum results cannot be achieved by simply classifying feature points and objects based on their depth. Therefore, this study adopts an adaptive depth-judgment strategy.

Following from Section 3.3.1, the straight lines formed by each point in the newPoints point set and their corresponding inward and outward expansion points are calculated. For each feature point located in the mask edge region, the nearest straight line is calculated using the equation

(6) $$d=\frac{\left(x_{e}-x_{r}\right)\left(y_{r}-y\right)-\left(y_{e}-y_{r}\right)\left(x_{r}-x\right)}{\sqrt{{\left(x_{e}-x_{r}\right)}^{2}+{\left(y_{e}-y_{r}\right)}^{2}}},$$

where d is the distance from the point to the corresponding straight line, $\left(x_{e},y_{e}\right)$ is a point in the expandPoints point set, $\left(x_{r},y_{r}\right)$ is the corresponding point in the reducePoints point set to $\left(x_{e},y_{e}\right)$, and $\left(x,y\right)$ is a feature point in the mask edge region.

**Figure 12:**
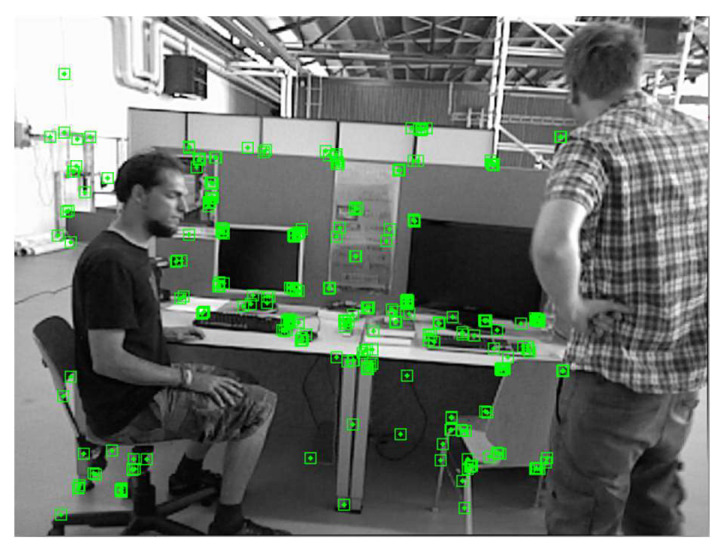
Effect of edge point rejection.

People can adopt different poses, and the depth values of different body parts can vary significantly. Therefore, for each feature point in the mask edge region, the inner feature point on the nearest straight line is selected. For the outer contour, the point in the reducePoints point set on the nearest straight line to the feature point is located. Then, its depth value is compared to that of the feature point to determine whether the point belongs to an obviously dynamic object, such as a person, or the background based on the threshold value. For the inner contour, the point in the expandPoints point set on the nearest straight line to the feature point is located. Then, the depth value is assessed. After debugging, the best effect is achieved when the threshold is set to 0.3 m. That is, when the difference between the depth of the feature point and the depth of the corresponding point is less than 0.3 m, the feature point is rejected; otherwise, it is retained. The rejection effect is shown in Figure 12.

#### 3.3.4. Potentially Dynamic Feature Point Rejection

In Section 3.3.1, Section 3.3.2 and Section 3.3.3, the feature points for obviously dynamic objects were successfully rejected. Semantic segmentation assumes that dynamic objects are moving constants. However, this may not apply to potentially dynamic objects, such as chairs; therefore, a different feature point rejection method is required. In this section, a feature point rejection strategy for potentially dynamic objects is proposed.

Potentially dynamic objects, such as chairs, often have more complex contours than obviously dynamic objects and they may be located in areas with low light. Therefore, the accuracy of the segmentation mask is greatly reduced if semantic segmentation is applied to potentially dynamic objects. Hence, this study uses a target detection method to assess internal feature points using detection box information. Subsequently, information about the feature points in the previous frame is used to ensure that each pair of feature points has a correct matching relationship and to determine whether a feature point is dynamic. Finally, based on the matching relationship, the epipolar geometry method is used to assess all feature points within the detection box and dynamic feature points are rejected.

**Figure 13:**
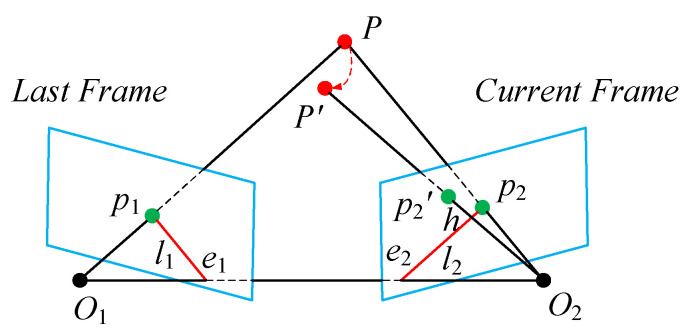
Diagram showing the epipolar geometry.

When the surrounding environment remains static, the matching points between two frames satisfy an epipolar geometric relationship. As shown in Figure 13, point P is a point in 3D space, and points $O_{1}$ and $O_{2}$ represent the camera position in frames 1 and 2, respectively. In addition, points $e_{1}$ and $e_{2}$ are the epipoles, lines $l_{1}$ and $l_{2}$ are the epipolar lines, and points $p_{1}$ and $p_{2}$ are the feature matching points in frames 1 and 2, respectively. Assume that the corresponding matching points between the previous frame feature points $p_{1}$ and the current frame feature points $p_{2}$ are

(7) $$p_{1}={\left[u_{1},v_{1},1\right]}^{T}$$

and

(8) $$p_{2}={\left[u_{2},v_{2},1\right]}^{T},$$

respectively, where $\left(u_{1},v_{1}\right)$ and $\left(u_{2},v_{2}\right)$ are the matching pixel coordinates of the previous and current frames. According to the definition of the fundamental matrix,

(9) $$P_{2}^{T}F_{21}p_{1}=0,$$

where $F_{21}$ denotes the fundamental matrix. Equation (9) describes the relationship between the coordinates of a static point in each frame. Fundamental matrix $F_{21}$ can be calculated as follows.

1. Find the feature points in the current frame that are not within the detection boxes (including all the detection boxes for obviously and potentially dynamic objects). These feature points are considered to be static and cannot move.
2. Find the feature points in the current frame that correspond to the feature points in the previous frame and establish a correct matching relationship.
3. Perform normalisation operations on the feature points that have established matching relationships.
4. Use the eight-point method to estimate the fundamental matrix.
5. Check the accuracy of the fundamental matrix.
6. Select the best matrix as the fundamental matrix based on the scores.

According to the definition of epipolar lines,

(10) $$l=F_{21}p_{1}={\left[\begin{array}{ccc} a_{2} & b_{2} & c_{2} \end{array}\right]}^{T},$$

where $a_{2}$, $b_{2}$, and $c_{2}$ are the coefficients of epipolar line l. If point P is a dynamic feature point, as shown in Figure 13, then point $P^{\prime }$ represents the point to which point P moves and its projection in the current frame is $p_{2}^{\prime }$. Owing to noise and other errors, a certain error exists between the theoretical and actual matching points for both dynamic and static feature points projected onto the current frame. The distance from point $p_{2}^{\prime }$ to the epipolar line $l_{2}$ is defined as h, where

(11) $$h=\frac{a_{2}u_{2}+b_{2}v_{2}+c_{2}}{\sqrt{a_{2}^{2}+b_{2}^{2}}}.$$

**Figure 14:**
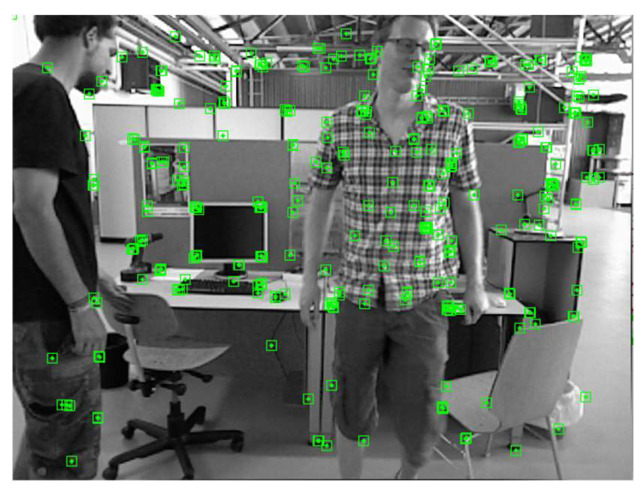
Effect of potentially dynamic feature point rejection. The feature points for the chair on the left are rejected.

For each pair of matching points, the corresponding error value h is calculated. By comparing the error value h with an empirical threshold, point P can be identified as a static or dynamic feature point. The rejection effect is shown in Figure 14, where the feature points for the chair on the left are rejected.

### 3.4. Dense 3D Mapping

Section 3.3 considered the effective elimination of dynamic feature points in dynamic scenes with the aim of improving the positioning accuracy of dynamic visual SLAM systems. By accurately identifying and eliminating the unstable feature points corresponding to dynamic objects, a solid foundation for subsequent map construction was established, which significantly improves the accuracy of dense mapping in dynamic scenes. This section further explores the application of dense-mapping technology to dynamic visual SLAM.

**Figure 15:**
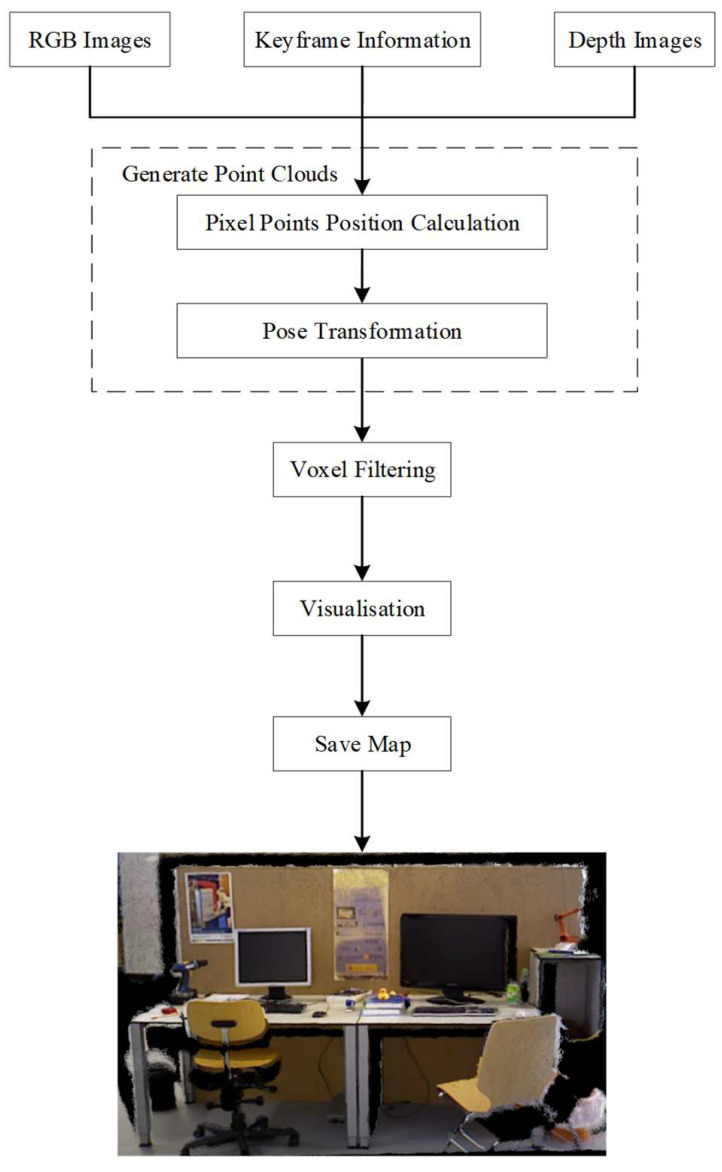
Flowchart showing the dense 3D mapping process.

Compared to sparse maps, dense maps have higher precision, more detailed representation, and better describe environmental structures, surface properties, and geometric shapes. Owing to their accuracy and detailed environmental representations, dense maps are widely used in fields such as robot navigation, environmental perception, and scene understanding. Advanced point cloud processing techniques are required to construct dense 3D mappings. The PCL library is one of the most popular open-source 3D point cloud libraries and it provides a wealth of algorithms and data structures, which makes making point-cloud processing relatively simple and efficient. This study constructs a static dense 3D mapping by combining information such as raw RGB images, depth maps, and keyframes. This process is summarised in Figure 15. First, the 3D coordinates and RGB colour values of each pixel in the keyframe images are calculated. Second, the coordinate pose is transformed into the global coordinate system. Third, when a new point cloud is added, voxel filtering operations are applied to the entire point cloud. Finally, the 3D dense mapping is output in the PCD format.

## 4. Experimental Results

In this section, the SEG-SLAM system will be demonstrated on the public TUM RGB-D datasets [38] and real scenes to evaluate its accuracy and robustness in dynamic scenes. The SEG-SLAM system was compared to the original ORB-SLAM3 system, DynaSLAM system, and other dynamic visual SLAM systems. The simulation experiments were conducted using a desktop computer with an Intel^®^ Core™ i5-9600KF CPU, 3.70 GHz clock speed, 16 GB RAM, NVIDIA GeForce GTX 1650 graphics card, and Ubuntu 20.04 operating system. The real-scene experiments were conducted using a Lenovo Y700 laptop with an Intel^®^ Core™ i5-6300HQ CPU, 2.30 GHz clock speed, 16 GB RAM, NVIDIA GeForce GTX 960M graphics card, and Ubuntu 18.04 operating system. For the semantic segmentation module, the input size of the RGB-D images was 640 × 640. For all the experiments, the feature point number *N* was 1000 and the depth threshold *d_t_* was 0.3 m.

The estimated camera trajectory data were compared to the real camera trajectory data using two common evaluation indicators: the absolute trajectory error (ATE) and relative pose error (RPE). The ATE describes the absolute error between the estimated and real poses, which can directly reflect the accuracy of the algorithm and the global consistency of the trajectory. The RPE describes the differences between the estimated and real poses in adjacent frames, including both rotation and translation errors. These indicators are crucial for assessing the accuracy and robustness of the system.

The root mean square error (RMSE) values for each indicator were used to evaluate the SEG-SLAM system. The RMSE of the ATE (ATE−RMSE) can be expressed as

(12) $$ATE-RMSE=\sqrt{\frac{1}{n}\sum_{i=1}^{n}Ⅱ\mathrm{trans}\left({\hat{E}}_{i}\right)-\mathrm{trans}{\left(T_{i}\right)}^{2}Ⅱ},$$

where ${\hat{E}}_{i}$ denotes the estimated camera pose, $T_{i}$ denotes the ground-truth pose, n is the number of frames in the image sequence, and trans denotes the transformation of the camera pose. In addition, the RMSE of the RPE (RPE−RMSE) can be expressed as

(13) $$RPE-RMSE=\sqrt{\frac{1}{m}\sum_{i=1}^{m}Ⅱ\mathrm{trans}{\left({\left(T_{i}^{-1}T_{i+\Delta }\right)}^{-1}\left({\hat{E}}_{i}^{-1}{\hat{E}}_{i+\Delta }\right)\right)}^{2}Ⅱ},$$

where Δ denotes the time interval, m denotes the difference between n and Δ. The experimental results were evaluated using the EVO evaluation tool and the results were used to produce the subsequent charts and other content.

### 4.1. TUM RGB-D Dataset

The TUM dataset is widely used to evaluate the robustness of indoor visual SLAM systems. It consists of data that were collected using Kinect RGB-D cameras with a frame rate of 30 Hz and a resolution of 640 × 480 pixels. The dataset was divided into 39 sequences based on the diversity of the scenes and motion states. For this study, eight sequences were selected from dynamic sequence fr3. Details of the selected sequences are given in Table 1. The sequences from the sitting and walking series datasets were lowly and highly dynamic sequences, respectively. They were integrated to ensure that the system was evaluated accurately and comprehensively for different dynamic scenarios.

#### 4.1.1. Comparison of the SEG-SLAM and ORB-SLAM3 Algorithms

**Figure 16:**
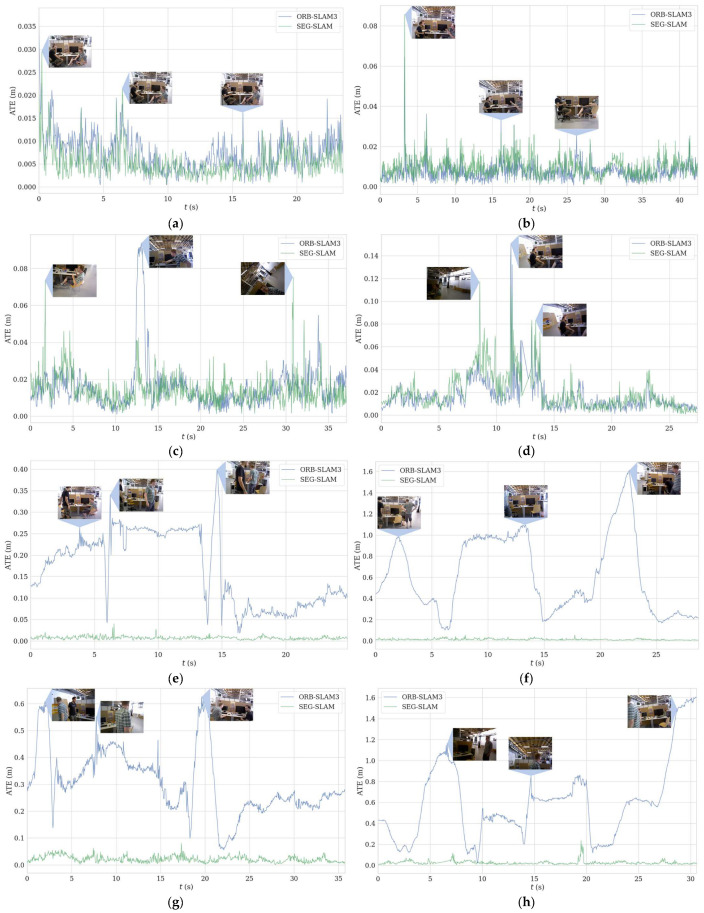
ATE distributions for the algorithms with different datasets. ATE distributions for the ORB-SLAM3 (blue) and SEG-SLAM (green) algorithms with sequences. (**a**) fr3/sitting_static, (**b**) fr3/sitting_xyz, (**c**) fr3/sitting_half, (**d**) fr3/sitting_rpy, (**e**) fr3/walking_static, (**f**) fr3/walking_xyz, (**g**) fr3/walking_half, and (**h**) fr3/walking_rpy. The RGB-D images represented by the blue lines in the figure indicate the dynamic objects in the scene.

The algorithm in this paper is improved based on ORB-SLAM3 [10]. To highlight the improvement, a detailed comparison between it and ORB-SLAM3 is conducted. The localisation performance of the two algorithms was evaluated by comparing their ATE−RMSE values, as shown in Table 2. SEG-SLAM exhibited greater precision than ORB-SLAM3 for the highly dynamic walking-series datasets, which indicates that it had superior performance for highly dynamic scenes. Furthermore, SEG-SLAM and ORB-SLAM3 produced similar results for the slightly dynamic sitting-series datasets. To visualise the differences in the precision of the two algorithms, the data in the table were plotted as shown in Figure 16.

**Table 2:** ATE−RMSE values for ORB-SLAM3 and SEG-SLAM with different sequences. The best values are indicated in bold.

	ATE−RMSE (m)	
Sequence	ORB-SLAM3	SEG-SLAM
fr3/sitting_static	0.0084	**0.0062**
fr3/sitting_xyz	**0.0088**	0.0110
fr3/sitting_half	0.0207	**0.0162**
fr3/sitting_rpy	**0.0221**	0.0252
fr3/walking_static	0.1882	**0.0081**
fr3/walking_xyz	0.7218	**0.0141**
fr3/walking_half	0.3283	**0.0243**
fr3/walking_rpy	0.7022	**0.0306**

The blue and green lines in Figure 16 represent the ATE values of the ORB-SLAM3 and SEG-SLAM algorithms, respectively. When dealing with the slightly dynamic sitting-series dataset, the curves for both algorithms showed similar changes. For the datasets where the camera moved in the *xyz* or *rpy* aspects, the ORB-SLAM3 algorithm showed superior overall performance. However, for the datasets where the camera was static or moved over a half sphere, the SEG-SLAM algorithm showed superior performance. This phenomenon can be explained by the fact that, for the slightly dynamic datasets, the small movements of the characters and discontinuities in the motion states made the ORB-SLAM3 algorithm more robust; thus, it performed better in specific scenarios. For the highly dynamic dataset, the movement characteristics of humans were more pronounced, which caused the ORB-SLAM3 algorithm to generate many erroneous matching feature points. Consequently, the camera pose was calculated incorrectly. By contrast, the SEG-SLAM algorithm uses a dynamic feature point rejection strategy. Consequently, only reliable static feature points were retained, which significantly reduced the number of feature point mismatches and increased the robustness of the algorithm. Therefore, the SEG-SLAM algorithm is more reliable for highly dynamic scenes and can provide valuable support for accurately reconstructing the camera trajectory.

#### 4.1.2. Comparison of the SEG-SLAM and DynaSLAM Algorithms

SEG-SLAM mainly uses semantic segmentation to obtain prior information about dynamic objects. To comprehensively demonstrate the robustness of the SEG-SLAM algorithm, it was compared to the classical DynaSLAM algorithm [27], which uses Mask-RCNN segmentation. Table 3 shows a detailed comparison of the results. SEG-SLAM showed better overall robustness than DynaSLAM and its ATE values were lower in each case except for the fr3/walking_static sequences. This indicates that, in most cases, SEG-SLAM performs better than DynaSLAM in terms of robustness and is more robust when dealing with dynamic scenes.

**Figure 17:**
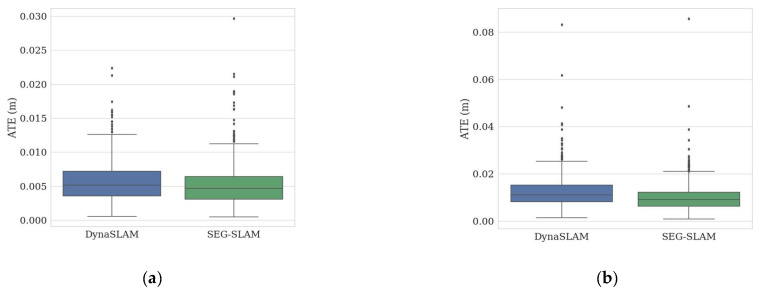

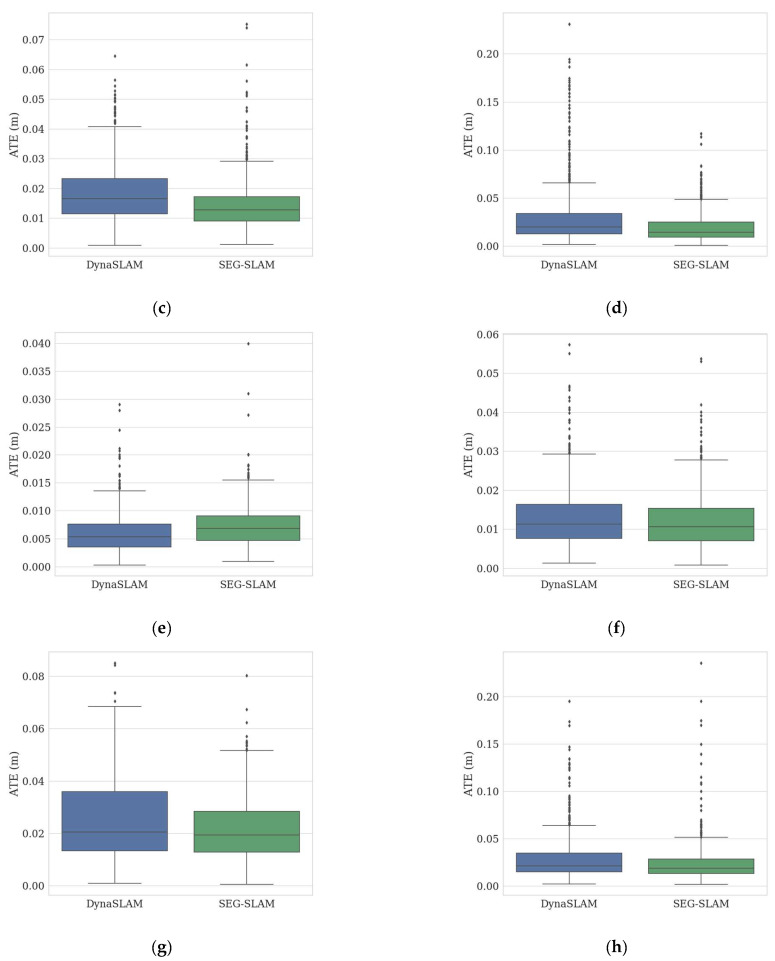
ATE distributions for the algorithms with different datasets. ATE distributions for the DynaSLAM (blue) and SEG-SLAM (green) algorithms with sequences (**a**) fr3/sitting_static, (**b**) fr3/sitting_xyz, (**c**) fr3/sitting_half, (**d**) fr3/sitting_rpy, (**e**) fr3/walking_static, (**f**) fr3/walking_xyz, (**g**) fr3/walking_half, and (**h**) fr3/walking_rpy.

To gain a deeper understanding of the differences between the two algorithms, box plots were used to conduct a comparative analysis of the ATE values, as shown in Figure 17. A box plot is a graphical representation of the distribution of data that provides a visual understanding of the centre of the data, the degree of dispersion, and outliers, which include medians, quartiles, outliers, and so on. SEG-SLAM had a lower median ATE than DynaSLAM for every dataset except fr3/walking_static, where it was slightly higher. This indicates that the SEG-SLAM algorithm generally performed better, although there were some exceptions. However, the number of exceptions was relatively small and they did not significantly affect the median value of the overall data distribution, which indicates that SEG-SLAM can produce accurate estimates in most cases. Further analysis of the exceptions will be conducted in the future to determine their causes and to identify relevant scenes or data features.

**Table 3:** ATE−RMSE values for DynaSLAM and SEG-SLAM with different sequences. The best results are indicated in bold.

	ATE−RMSE (m)	
Sequence	DynaSLAM	SEG-SLAM
fr3/sitting_static	0.0065	**0.0062**
fr3/sitting_xyz	0.0136	**0.0110**
fr3/sitting_half	0.0209	**0.0162**
fr3/sitting_rpy	0.0441	**0.0252**
fr3/walking_static	**0.0071**	0.0081
fr3/walking_xyz	0.0151	**0.0141**
fr3/walking_half	0.0291	**0.0243**
fr3/walking_rpy	0.0371	**0.0306**

#### 4.1.3. Comparison of Mainstream Dynamic Visual SLAM Algorithms

To better demonstrate the robustness of the SEG_SLAM algorithm, several recent mainstream dynamic visual SLAM algorithms were selected for comparison. DynaSLAM uses the Mask-RCNN method to eliminate dynamic feature points, DS-SLAM [39] uses the SegNet method to eliminate dynamic feature points, and RDS-SLAM [40] uses both the Mask-RCNN and SegNet methods to eliminate dynamic feature points. Dynamic-VINS [41] uses target detection and depth information fusion in resource-limited environments to eliminate dynamic feature points, and YOLO-SLAM [29] uses an improved YOLOv3 neural network to eliminate dynamic feature points. Table 4 shows the ATE−RMSE for the various algorithms. The data for DynaSLAM and DS-SLAM are the actual test results, whereas the data for the other algorithms were obtained from the relevant references. SEG-SLAM showed the best results in six cases, and the ATE−RMSE only exceeded those of the other algorithms for the fr3/sitting_rpy and fr3/walking_static sequences. The segmentation algorithms used by DynaSLAM and DS-SLAM have higher precision; therefore, they may produce better results for individual static datasets.

Table 5 and Table 6 show the relative errors in the pose transformation for various dynamic visual SLAM algorithms. The SEG-SLAM algorithm had the best RMSE values for four sequences and the differences between the RMSE values for the SEG-SLAM algorithm, and the best results were small for the other sequences. Therefore, SEG-SLAM has a significant advantage in terms of the translation and rotation errors compared to other dynamic visual SLAM algorithms.

**Figure 18:**
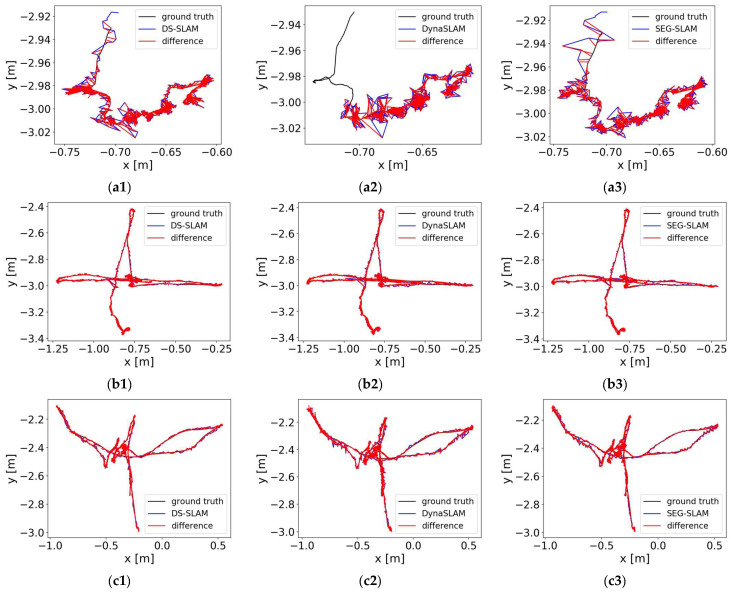

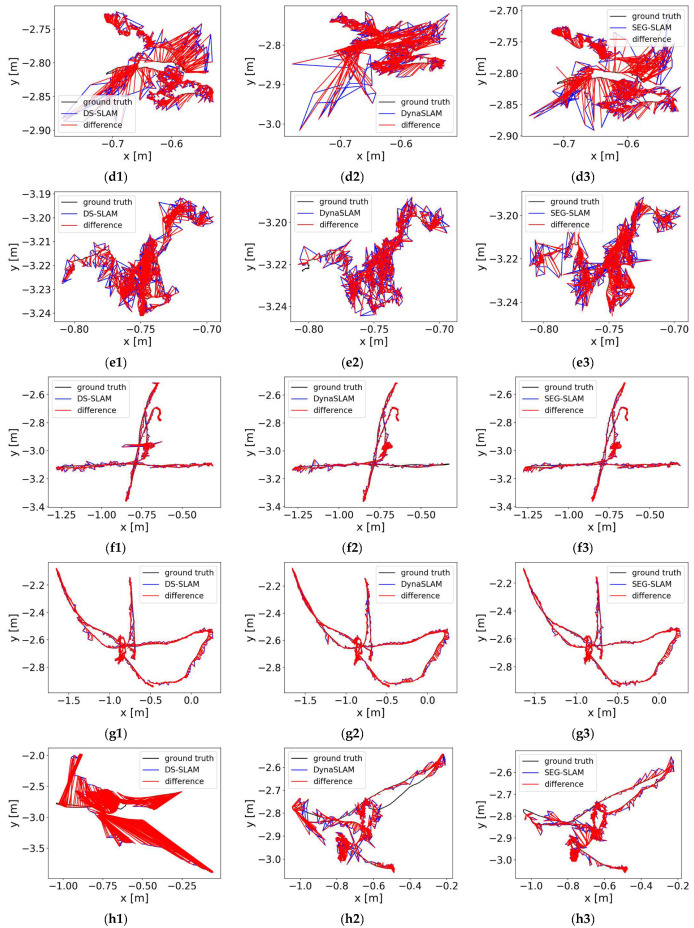
Camera trajectory errors of different methods. The black line represents the ground truth of the camera trajectory, the blue line represents the estimated trajectory, and the red line represents the absolute difference between the estimated trajectory and the ground truth. The results are presented for sequences (**a**) fr3/sitting_static, (**b**) fr3/sitting_xyz, (**c**) fr3/sitting_half, (**d**) fr3/sitting_ rpy, (**e**) fr3/walking_static, (**f**) fr3/walking_ xyz, (**g**) fr3/walking_ half, and (**h**) fr3/walking_ rpy and algorithms (**1**) DS-SLAM, (**2**) DynaSLAM, and (**3**) SEG-SLAM.

To compare the overall motion error of the camera more intuitively, the differences between the estimated camera trajectories and the ground truths are illustrated in Figure 18. Among the eight sequences, the trajectory errors for the three algorithms were similar. However, SEG-SLAM had lower overall trajectory errors and was more robust than the other algorithms.

**Table 5:** Comparison of RPE−RMSE values for various algorithms with different sequences (RPE translation part). Here, “-“ indicates that there were no relevant experimental data, and the best results are indicated in bold.

	RPE−RMSE (m/s)					
Sequence	DynaSLAM	DS-SLAM	RDS-SLAM	Dynamic-VINS	YOLO-SLAM	SEG-SLAM
fr3/sitting_static	0.0055	**0.0045**	0.0097	-	0.0089	0.0055
fr3/sitting_xyz	0.0106	**0.0088**	-	-	-	0.0096
fr3/sitting_half	0.0163	**0.0101**	-	-	-	0.0124
fr3/sitting_rpy	0.0216	0.0149	-	-	-	**0.0148**
fr3/walking_static	0.0070	**0.0052**	0.0529	0.0095	0.0094	0.0070
fr3/walking_xyz	0.0124	0.0146	0.0299	0.0578	0.0194	**0.0112**
fr3/walking_half	0.0149	0.0134	0.0332	0.0665	0.0268	**0.0133**
fr3/walking_rpy	0.0271	0.0230	0.0700	0.0595	0.0933	**0.0200**

**Table 6:** Comparison of RPE−RMSE values for various algorithms with different sequences (RPE rotation part). Here, “-“ indicates that there were no relevant experimental data, and the best results are indicated in bold.

	RPE−RMSE (deg/s)					
Sequence	DynaSLAM	DS-SLAM	RDS-SLAM	Dynamic-VINS	YOLO-SLAM	SEG-SLAM
fr3/sitting_static	0.0040	**0.0038**	0.3217	-	0.2709	0.0041
fr3/sitting_xyz	0.0082	**0.0078**	-	-	-	0.0079
fr3/sitting_half	0.0114	**0.0097**	-	-	-	0.0100
fr3/sitting_rpy	0.0117	0.0108	-	-	-	**0.0102**
fr3/walking_static	0.0046	**0.0041**	1.4966	0.4581	0.2623	0.0046
fr3/walking_xyz	0.0097	0.0105	0.7739	1.6932	0.5984	**0.0094**
fr3/walking_half	0.0106	0.0105	0.8194	5.2116	0.7534	**0.0102**
fr3/walking_rpy	0.0147	0.0134	1.4736	5.0839	1.8238	**0.0126**

#### 4.1.4. Dense 3D Mapping

**Figure 19:**
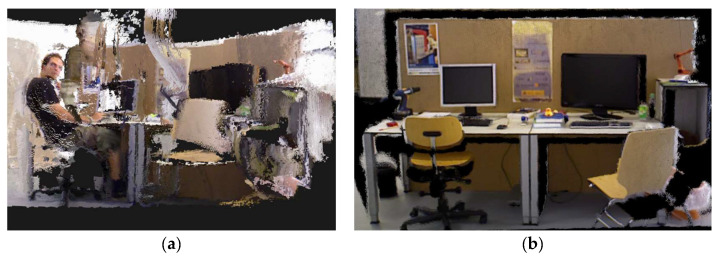
Dense 3D mapping. Results obtained using (**a**) ORB-SLAM3 and (**b**) SEG-SLAM.

SEG-SLAM was used to generate a static dense 3D mapping by eliminating dynamic objects from the scene. The process was tested using the TUM dataset and the results are shown in Figure 19. Figure 19a shows the result obtained using ORB-SLAM3, which did not remove the dynamic objects and contains a lot of dynamic object ghosting. Figure 19b shows the result obtained using SEG-SLAM, which does not contain obviously dynamic objects.

### 4.2. Real Scene

**Figure 20:**
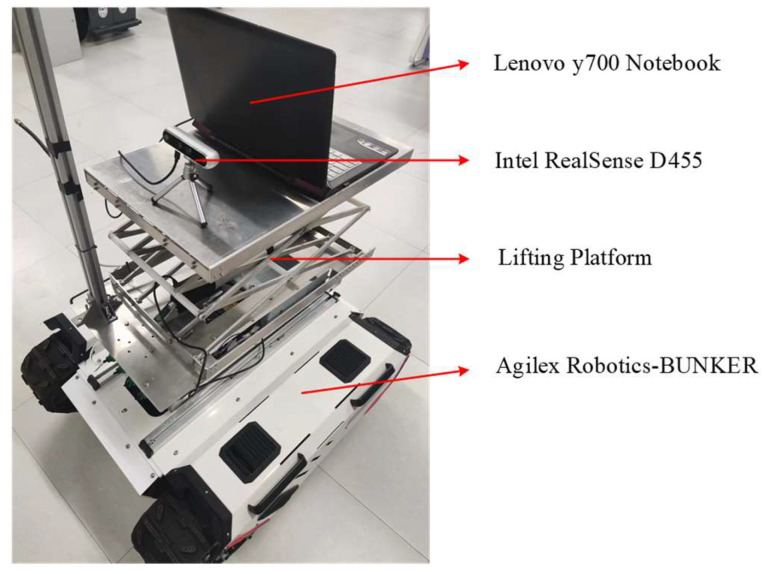
Photograph of the experimental platform.

The robustness of the SEG-SLAM system with real scenes was verified using the experimental platform shown in Figure 20. A Lenovo y700 laptop (Lenovo, Beijing, China) and Intel RealSense D455 depth camera (Intel, Santa Clara, CA, USA) were positioned on the lifting platform of an Agilex Robotics-BUNKER mobile platform (AgileX Robotics, Shenzhen, China).

**Figure 21:**
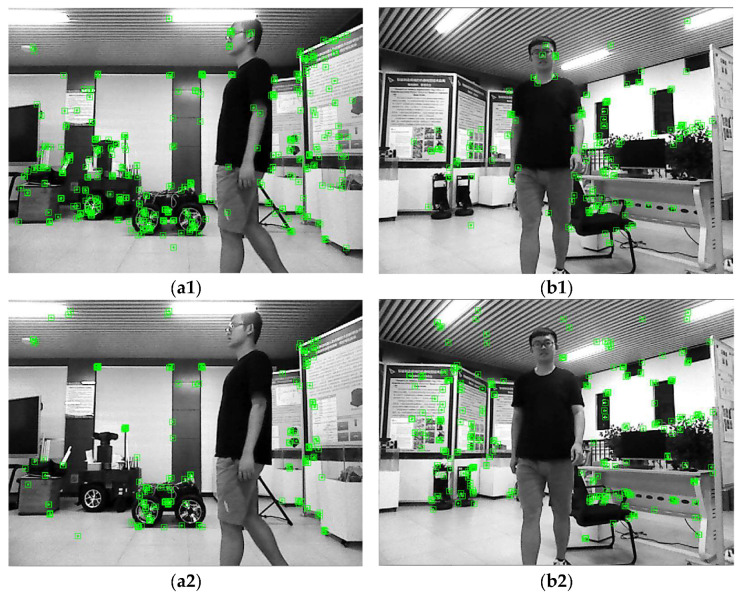
Images showing actual dynamic scene effects. Results for two perspectives (**a**,**b**) obtained using (**1**) ORB-SLAM3 and (**2**) SEG-SLAM.

**Figure 22:**
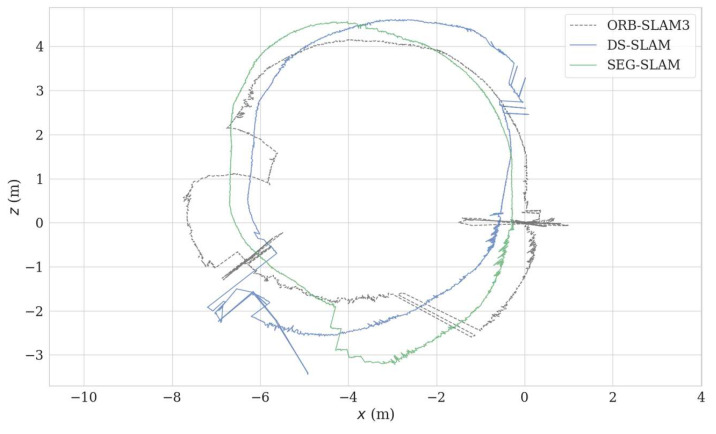
Comparison of the circular trajectories obtained for an indoor dynamic scene using various algorithms.

The experimental scene consisted of an indoor office area where humans were the main moving objects. The mobile robot travelled along a circular route. As shown in Figure 21, two perspectives were used to compare the results of ORB-SLAM3 and SEG-SLAM. ORB-SLAM3 failed to eliminate dynamic object feature points, whereas SEG-SLAM eliminated feature points effectively and did not mistakenly eliminate static background feature points. Figure 22 shows a comparison of the trajectories obtained via ORB-SLAM3, DS-SLAM, and SEG-SLAM. SEG-SLAM had the best trajectory effect, whereas ORB-SLAM3 caused serious trajectory drifting owing to erroneous data associations between matching points caused by the retained dynamic feature points. The trajectories obtained using DS-SLAM and SEG-SLAM showed similar trajectories. However, DS-SLAM showed local drifting. Owing to the unstable structure of the lifting platform, the camera shook as the robot moved, which resulted in local trajectory jitters.

### 4.3. Running Time Analysis

The running time is an important consideration when evaluating visual SLAM systems. The median and mean tracking times for each frame were used to evaluate the running efficiency of SEG-SLAM compared to other algorithms, as shown in Table 7 and Table 8. The TUM dataset was tested using a desktop computer and the real scene was tested on a laptop. For the TUM dataset, SEG-SLAM has a higher operating efficiency than DynaSLAM and a lower operating efficiency than DS-SLAM. For the real scene, SEG-SLAM and DS-SLAM had similar operating efficiencies. Therefore, when considering semantic segmentation methods to obtain prior information about objects, SEG-SLAM exhibits high robustness, although its overall operating efficiency is relatively low. After the analysis, YOLOv5 was found to have high computational requirements when calculating the object mask information, which affected the real-time performance of the system. In the future, lightweight operations will be applied to the YOLOv5 segmentation process to improve the running speed of the visual SLAM system.

## 5. Discussion

This study proposed an RGB-D SLAM system that integrated geometric and semantic information, resulting in remarkable robustness for dynamic environments. Conventional dynamic visual SLAM systems based on geometric information ignore potentially dynamic objects, whereas those based on semantic information struggle to meet real-time performance requirements. Therefore, this study used a target detection and semantic segmentation fusion module based on YOLOv5. Target detection can only obtain the boundary information of objects, which is prone to incorrectly eliminating points from the background. Semantic segmentation can effectively identify object contour information, although it is highly dependent on the mask quality. Therefore, the YOLOv5 neural network was used to combine both types of information. In the feature point elimination module, if only the internal points of obviously dynamic objects are eliminated, some errors will occur. Therefore, an adaptive strategy for dynamic feature point selection was designed, and the epipolar geometry method was used to eliminate the feature points of potentially dynamic objects.

To evaluate the reliability of the system, the performance of the SEG-SLAM system was compared to various mainstream dynamic visual SLAM systems using the TUM dataset and real scenes. The experimental results are presented in Table 2, Table 3, Table 4, Table 5 and Table 6. The positioning accuracy of SEG-SLAM was higher than that of ORB-SLAM3 for highly dynamic sequences and higher than or similar to that of ORB-SLAM3 for slightly dynamic sequences. The ATE values of the two algorithms were compared graphically, as shown in Figure 16. SEG-SLAM was also compared to the state-of-the-art DynaSLAM system, which uses the Mask-RCNN semantic segmentation method. SEG-SLAM showed better overall positioning accuracy and real-time performance than DynaSLAM. The differences between the results obtained using the two algorithms were compared using box plots, as shown in Figure 17. SEG-SLAM used a target detection and semantic segmentation fusion module based on YOLOv5, which had a higher positioning accuracy than other mainstream algorithms based on SegNet, Mask-RCNN, and YOLOv3 target detection. In real scenarios, this paper’s algorithm has higher accuracy compared to ORB-SLAM3 and DS-SLAM algorithms. In terms of real-time, the algorithm in this paper has higher real-time compared to DynaSLAM, DS-SLAM, and other SLAM systems using semantic segmentation algorithms.

## 6. Conclusions

This study proposed a robust dynamic visual SLAM system based on ORB-SLAM3. To prevent erroneous data associations caused by dynamic objects in a scene, a target detection and semantic segmentation fusion module based on YOLOv5 was developed. In this module, target detection was used to detect potentially dynamic objects such as chairs and output the object bounding boxes and category information. Semantic segmentation was used to detect obviously dynamic objects and output the object mask and category information. A differentiated dynamic feature point elimination strategy was developed using the dynamic object prior information, depth information, and epipolar geometry. First, for obviously dynamic objects, the mask information was used to eliminate internal feature points. Second, after considering the error of the mask contour, the edge feature points of obviously dynamic objects were eliminated. Finally, for potentially dynamic objects, the epipolar geometry method was used to eliminate dynamic feature points. In addition, keyframes with eliminated dynamic feature points were combined with the PCL library to build a static dense 3D mapping. The SEG-SLAM system was evaluated using the challenging TUM RGB-D dataset and real scenes, and the results were compared to those obtained using the most advanced dynamic visual SLAM algorithms reported in recent years. The results showed that the SEG-SLAM system effectively processes the mask edge feature points and rejects the dynamic feature points of potentially dynamic objects, has higher localisation accuracy than mainstream dynamic vision SLAM systems, and has higher real-time performance in dynamic vision SLAM systems using semantic segmentation algorithms, so the algorithm in this paper has higher accuracy and robustness in dynamic environments.

Although the algorithm in this paper has achieved excellent performance, there are still some problems in the following two aspects: (1) compared with the target detection to obtain semantic information methods, the algorithm in this paper has a large amount of computation, and the real-time performance is on the low side; (2) the algorithm of this paper has some limitations when obviously dynamic objects are not in motion at a certain moment. In the future, several aspects of the SEG-SLAM system should be optimised. First, to improve the real-time performance, distillation methods may be used to develop lightweight YOLOv5 models, and more reasonable multithreading methods may be used to deploy the target detection and semantic segmentation modules. Second, for obviously dynamic objects that are stationary at a given moment, reasonable strategies should be designed to recover the feature point information within the mask. Finally, for dense mapping, filtering methods should be used to further optimise the map quality.

## Figures and Tables

**Table 1:** Details of the selected sequences.

Sequence	Duration (s)	Trajectory Length (m)	Number of Frames	Camera Movement
fr3/sitting_static	23.63	0.259	680	Static
fr3/sitting_xyz	42.50	5.496	1219	Along *xyz*
fr3/sitting_half	37.15	6.503	1074	Along half sphere
fr3/sitting_rpy	27.48	1.110	795	Along *rpy*
fr3/walking_static	24.83	0.282	717	Static
fr3/walking_xyz	28.83	5.791	827	Along *xyz*
fr3/walking_half	35.81	7.686	1021	Along half sphere
fr3/walking_rpy	30.61	2.698	866	Along *rpy*

**Table 4:** Comparison of the ATE−RMSE values for various algorithms with different sequences. Here, “-“ indicates that there were no relevant experimental data, and the best results are indicated in bold.

	ATE−RMSE (m)					
Sequence	DynaSLAM	DS-SLAM	RDS-SLAM	Dynamic-VINS	YOLO-SLAM	SEG-SLAM
fr3/sitting_static	0.0065	0.0072	0.0084	-	0.0089	**0.0062**
fr3/sitting_xyz	0.0136	0.0111	-	-	-	**0.0110**
fr3/sitting_half	0.0209	0.0170	-	-	-	**0.0162**
fr3/sitting_rpy	0.0441	**0.0243**	-	-	-	0.0252
fr3/walking_static	**0.0071**	0.0080	0.0720	0.0077	0.0094	0.0081
fr3/walking_xyz	0.0151	0.0191	0.0240	0.0486	0.0194	**0.0141**
fr3/walking_half	0.0291	0.0299	0.0306	0.0608	0.0268	**0.0243**
fr3/walking_rpy	0.0371	0.4352	0.0587	0.0629	0.0933	**0.0306**

**Table 7:** Comparison of computation times for different algorithms with TUM (fr3/walking_xyz). The best results are indicated in bold.

	Computation Time (s)	
System	Median	Mean
DynaSLAM	0.2771	0.5655
DS-SLAM	**0.0544**	**0.0643**
SEG-SLAM	0.1168	0.1189

**Table 8:** Comparison of computation times for different algorithms with real scenes. The best values are indicated in bold.

	Computation Time (s)	
System	Median	Mean
ORB-SLAM3	0.0215	0.0222
DS-SLAM	**0.0561**	**0.0609**
SEG-SLAM	0.0617	0.0687

## Data Availability

The dataset used in this paper is the public TUM dataset. The download address is as follows: https://vision.in.tum.de/data/datasets/rgbd-dataset (accessed on 21 March 2024).
